# Epidemiological and clinical profile of hypertensive octogenarian patients and factors associated with uncontrolled hypertension: observational study of 346 patients

**DOI:** 10.11604/pamj.2021.39.202.28466

**Published:** 2021-07-15

**Authors:** Amine Bahloul, Rania Hammami, Selma Charfeddine, Sirine Triki, Nadia Bouattour, Leila Abid, Samir Kammoun

**Affiliations:** 1 Hedi Chaker University Hospital, Sfax, Tunisia,; 2 Habib Bourguiba University Hospital, Sfax, Tunisia

**Keywords:** Blood pressure, elderly, uncontrolled hypertension, pulse pressure, risk factor

## Abstract

**Introduction:**

hypertension (HTN) is the main risk factor for most morbidities of elderly subjects. The objective of this study was to identify the epidemiological and clinical characteristics of hypertension in octogenarians and to identify the factors associated with uncontrolled hypertension in this population.

**Methods:**

we used data collected in the outpatient cardiology department of the University Hospital of Sfax between 15^th^ April 2019 and 15^th^ May 2019 as part of the National Tunisian Registry of Hypertension. We included in our study patients aged 80 years or more with hypertension. We described the epidemiological and clinical profile of this population, and we studied the associations between uncontrolled hypertension and socio-demographic, lifestyle, clinical and therapeutic factors using logistic regression models.

**Results:**

we included 346 subjects (45.1% (n=156) male and 54.9% (n=190) female), with a mean age of 84.36 (SD 4.01) years. More than half of them had uncontrolled hypertension. Dyslipidemia was the most common cardiovascular risk factor found in 43.6 % (n=151) of patients followed by diabetes (35.5%, n=122). One-third of patients had a history of coronary artery disease and/or stroke. Renal failure and kalemia disorders were observed, respectively, in 12.1% (n=42) and 25.2% (n=40) of patients. In multivariate analysis, factors associated with uncontrolled hypertension (HTN) were male sex (adjusted odds ratio (aOR): 1.663, 95% confidence interval (CI): 1.045-2.647; p=0.032), diabetes (aOR: 1.66, 95%CI: 1.031-2.688; p=0.037,) and poor adherence to blood pressure (BP) medications (aOR: 1.960, 95%CI: 1.195-3.214; p=0.008).

**Conclusion:**

our results showed that more than half of octogenarian hypertensive patients did not reach the BP target and that poor adherence to BP medications was the main factor of uncontrolled HTN. In this population, the presence of other comorbidities and poor adherence to BP medications are very common. Systematic research for behaviors suggesting poor medication adherence should be a priority for physicians caring for these patients.

## Introduction

Hypertension (HTN) is one of the most common morbidities and the main risk factor for most morbidities of elderly subjects, with a significant impact on their prognosis [1]. Large studies, like the Framingham Heart Study, have shown that HTN is closely linked to increased morbidity and mortality in the elderly [2]. Africa, whether in the Maghreb or in sub-Saharan countries, is not spared by this global health problem, with an annual hypertension mortality rate estimated at around 150 per 100,000 inhabitants, for a world average of 125 per 100,000 inhabitants [3,4]. The variability in the presentation and the evolution of HTN makes it a complicated disease, which requires optimal control and persistent drug-adherence [1]. The objective of this study was to identify the epidemiological and clinical characteristics of HTN in Tunisian octogenarians and to identify the factors associated with uncontrolled HTN in this population.

## Methods

**Study design and settings:** we used data collected in the outpatient cardiology department of the University Hospital of Sfax between 15^th^ April 2019 and 15^th^ May 2019 as part of the National Tunisian Registry of Hypertension (NATURE HTN). The NATURE HTN registry is a national, observational, cross-sectional and multi-center study planned by the Tunisian Society of Cardiology and Cardiovascular Surgery and validated by an ethics committee. It was designed by a multidisciplinary committee of cardiologists from different hospital structures in Tunisia. The main objectives of this registry were to describe the epidemiological profile of HTN in Tunisia and to assess the level of cardiovascular risk of hypertensive Tunisian patients.

**Study population:** our study through the data we analyzed aimed to describe the epidemiological and clinical profile of octogenarian hypertensive patients and determine factors associated with uncontrolled hypertension in this population. The inclusion criteria were an age greater or equal to 80 with a confirmed essential HTN. Patients with short life expectancy and end-stage kidney failure with chronic hemodialysis were excluded.

### Data collection

*Blood pressure (BP):* systolic BP (SBP) and diastolic (DBP) was measured using a manual or automated electronic sphygmomanometer in accordance with the guidelines of the European Society of Cardiology (ESC) [5] on the right arm using a cuff adapted to arm circumference. The measurements were performed after 15 minutes of rest, with a patient seated, and the arm placed in the appropriate position. Three measurements were taken 1 to 2 minutes apart. The SBP and DBP retained for each person correspond to the average of the last two measures. Subjects who did not have at least two BP readings were excluded from the analysis. We considered that the HTN is uncontrolled if the BP is not at the target, which is a SBP less than 140 mmHg and a DBP less than 90 mmHg [5]. From the values of SBP and DBP we calculated the pulse pressure (PP) which is the difference between the SBP and the DBP.

*Covariates:* we collected, through a questionnaire, demographic data (age and sex), weight and height with calculation of the body mass index (BMI), lifestyle behavior (adherence or not to a low sodium diet, the practice or not of a physical activity, smoking status, cardiovascular risk factors (dyslipidemia, diabetes), history of cardiovascular and cerebrovascular diseases (coronary artery disease, stroke) and therapeutic data (number and classes of anti-hypertensive drugs namely angiotensin converting enzyme (ACE), angiotensin receptor blocker (ARB), calcium channel blocker (CCB), diuretic and beta blocker (BB). The adherence to treatment was assessed using the Girerd score [6]. Patients were classified as poor adherents if the Girerd score was equal to or greater than 3, and as good adherents if the Girerd score was less than 3. The presence or absence of Atrial Fibrillation (AF) was investigated by questioning, clinical examination and an electrocardiography (ECG). Presence or absence of renal failure, defined by a clearance of creatinine < 60 milliliters per minutes (mL/mn) [7] and calculated according to the Modification of Diet in Renal Disease (MDRD) formula, was conducted on the basis of the last creatinine value reported by the patient and dating back less than 6 months. Kalemia was noted only in patients who had reported a recent blood ionogram of less to 1 month. The search for a left ventricular hypertrophy (LVH) was performed on an ECG of less 6 months (defined by a Sokolow Index ≥ 35 millimeter (mm) and on the last echocardiography done in less than 12 months (defined by a left ventricular mass of 100 g/m^2^ in women and 115g/m^2^ in men) [8]. Regarding the grouping: we divided smoking status into 2 groups (current smoker or no current smoker); we categorized BMI into two groups: < 30 kg/m^2^ as non-obese, and ≥ 30 kg/m^2^ as obese; we divided kalemia into 3 groups using the serum potassium standards of the hospital laboratory, indicated between 3.5 and 4.5 mmol/l.

**Statistical analysis:** the data collected were analyzed using IBM SPSS Statistics version 23.0 for Windows (Chicago, Illinois, USA). We first performed a descriptive analysis comparing the two sexes. We then performed univariate analysis followed by multivariate analysis to investigate the relationship between uncontrolled HTN (BP ≥ 140/90 mmHg) and the different variables using logistic regression models. Furthermore, we have included in the multivariate statistical model all variables with p-value < 0.25 in the univariate analysis. A p-value <0.05 was considered significant.

**Ethical considerations:** the NATURE HTN registry was conducted after obtaining approval from the ethics committee of the Tunis internal security forces hospital. The ethical aspects were respected. Informed patient consent was required for all study participants. Participation in the study was free. Confidentiality and anonymity were guaranteed to patients who participated in the study.

## Results

**Epidemiological, clinical and paraclinical characteristics of the population:** we included 346 subjects (45.1% (n=156) male and 54.9% (n=190) female), with a mean age of 84.36 (SD 4.01) years. More than half of the patients (51.4%, n=178) had uncontrolled HTN. Nearly a third of patients (32.2%, n=110) had systolic isolated HTN and 57% of patients had an increase in PP (≥60 mmHg). Dyslipidemia was the most common cardiovascular risk factor found in 43% (n=151) of subjects with a significant male predominance (51.3% (n=80) versus 37.4% (n=71), p <0.009), followed by diabetes present in more than one-third of subjects with equal distribution between the two sexes. One-third of patients had a history of coronary artery disease and/or stroke, and 14.2% of patients had a history of atrial fibrillation. The mean value of creatinine was 96.31(SD 42.70) mmol/l. Renal failure was noted in 12.1% (n=42) of patients. In addition, kalemia disorders were present in 25.2% (n=40) of patients and were, for the most part, a hyperkalemia (20.8%, n=33). The socio-demographic, clinical and paraclinical characteristics of the population are summarized in Table 1 and the blood pressure status is summarized in Table 2.

**Table 1:** general population characteristics

				Sex		p value	Total
				Men	Women		
Clinical features	Mean age		Mean (SD)	84.21/-3.49	84.48/-4.39	0.53	84.36/-4.01
	Mean BMI		Mean (SD)	25.82/-3.41	27.88/-4.81	<0.001	26.95/-4.35
	Diabetes		N (%)	61 (39.1)	61 (31.1)	0.175	122 (35.5)
	Dyslipidemia		N (%)	80 (51.3)	71 (37.4)	0.009	151(43.6)
	Obesity		N (%)	16 (10.3)	57 (30.0)	<0.001	73 (21.1)
	Stroke		N (%)	14 (9)	26 (13.7)	0.173	40 (11.6)
	Coronary artery disease		N (%)	48 (30.8)	30 (15.8)	0.001	78 (22.5)
	AF		N (%)	21 (13.5)	28 (14.7)	0,735	49 (14.2)
Paraclinical features	LVH on ECG		N (%)	23 (14.7)	45 (23.7)	0.037	68 (19.7)
	LVH on cardiac ultrasound		N (%)	35 (22.4)	49 (25.8)	0,469	84 (24.3)
	Creatinine (mmol/l)		(Mean/SD)	102.39/-29.03	90.66/-51.81	0.061	96.31/-(42.70)
	Renal failure		N (%)	23 (14.7)	19 (10)	0.179	42 (12.1)
	Kalemia	3.5 mmol/L	N (%)	1 (1.4)	6 (7)	0.186	7 (4.4)
		3.5-4.9 mmol/l	N (%)	58 (79.5)	61 (70.9)		119 (74.8)
		≥ 5 mmol/l	N (%)	14 (19.2)	19 (22.1)		33 (20.8)
Hygienic-dietary characteristics	Smoking		N (%)	23 (14.7)	1 (0.5)	< 0.001	24 (6.9)
	No physical activity		N (%)	140 (81.7)	179 (94.2)	0.123	319 (92.2)
	Poor adherence to BP medication		N (%)	67 (42.9)	76(40.0)	0.579	143 (41.3)
	Non-compliance with low sodium diet		N (%)	25 (16)	20 (10.5)	0.13	45 (13.0)

AF: atrial fibrillation, BMI: body mass index, BP: blood pressure, ECG: electrocardiogram LVH: left ventricular hypertrophy, N (%): number and percentage of patients, SD: standard deviation

**Table 2:** blood pressure status and anti-hypertensive treatment

			Men	Women	p value	Total
SBP		(Mean/SD)	142.32/-20.06	136.24/-19.24	0.005	138.98/-19.81
DBP		(Mean/SD)	79.07/-13.76	75.85/-10.36	0.014	77.29/-12.09
PP		(Mean/SD)	63.34/-18.06	60.43/-15.20	0.119	61.69/-16.57
BP ≥140/90 mmHg		N (%)	67 (42.9)	111 (58.4)	0.005	178 (51.4)
SBP≥ 140 mmHg		N (%)	87 (56.9)	78 (41.3)	0.004	165 (48.2)
DBP ≥90 mmHg		N (%)	38 (24.8)	20 (10.6)	0.001	58 (17)
PP≥60 mmHg		N (%)	92 (60.1)	103 (54.5)	0.001	195 (57)
Grade of HTN	HTN grade I	N (%)	57 (36.5)	53 (27.9)	0.065	110 (31.8)
	HTN grade II	N (%)	20 (12.8)	16 (8.4)		36 (10.4)
	HTN grade III	N (%)	12 (7.7)	10 (5.3)		22 (6.4)
	Isolated systolic HTA	N (%)	51 (33.3)	59 (31.2)	0.668	110 (32.2)
Number of anti-hypertensive drugs	No medication	N (%)	17(10.9)	15(7.9)	0.554	32 (9.2)
	Monotherapy	N (%)	78 (50.0)	88 (46.3)		48 (52)
	Bitherapy	N (%)	43 (27.6)	59 (31.1)		102 (29.5)
	Tritherapy	N (%)	18 (11.5)	28 (14.7)		46 (13.3)
ACE		N (%)	72 (46.2)	98 (52.6)	0.315	170 (49.1)
ARB		N (%)	20 (12.8)	28 (14.7)	0.608	48 (13.9)
CCB		N (%)	59 (37.8)	80 (42.1)	0.419	139 (40.2)
Diuretic		N (%)	16 (10.3)	26 (13.7)	0.331	42 (12.1)
BB		N (%)	35 (22.4)	38 (20.0)	0.598	73 (21.1)

ACE: angiotensin converting enzyme, ARB: angiotensin receptor blockers, BB: beta blockers, CCB: calcium channel blockers, DBP: diastolic blood pressure, HTN: hypertension, N(%): number and percentage of patients, PP: pulse pressure, SBP: systolic blood pressure, SD: Standard deviation

**Factors associated with uncontrolled hypertension:** in univariate analysis (Table 3), factors associated with uncontrolled HTN were male sex, diabetes, non-compliance with the low sodium diet and poor adherence to BP medications. In multivariate analysis (Table 3), factors associated with uncontrolled HTN were male sex (Adjusted Odds ratio (aOR): 1.663, 95% Confidence interval (CI): 1.045-2.647; p=0.032), diabetes (aOR: 1.66, 95%CI: 1.031-2.688; p=0.037,) and poor adherence to blood pressure medications (aOR: 1.960, 95%CI: 1.195-3.214; p=0.008). However, none of the following factors were significantly associated with uncontrolled HTN: obesity, smoking, dyslipidemia, left ventricular hypertrophy (LVH), cardiovascular or kidney disease, class of BP medication and number of anti-hypertensive drugs.

**Table 3:** univariable and multivariable analysis of factors associated with uncontrolled hypertension

	Univariable analysis		Multivariable analysis	
	OR (95%CI)	p value	OR (95%CI)	p-value
Male sex	1.866 (1.216-2.865)	0.004	1.663 (1.045-2.647)	0.032
Diabetes	0.608 (0.390-0.949)	0.028	1.66 (1.031-2.688)	0.037
Dyslipidemia	0.765 (0.500-1.172)	0.218	0.815 (0.512-1.299)	0.390
Obesity (BMI> 30Kg/m^2^)	0.781 (0.465-1.311)	0.349		
LVH on ECG	0.747 (0.439-1.271)	0.281		
LVH on cardiac ultrasound	0.720 (0.439-1.179)	0.191	0.761 (0.448-1.293)	0.313
Stroke	1.049 (0.542-2.029)	0.887		
coronary heart disease	0.928 (0.560-1.537)	0.772		
Atrial fibrillation	1.440 (0.780-2.66)	0.242	1.682 (0.877-3.225)	0.118
Renal failure	0.675 (0.352-1.295)	0.235	0.734 (0.370-1.456)	0.376
No physical activity	1.196 (0.543-2.636)	0.665		
Smoking	0.447 (0.186-1.074)	0.066	0.753 (0.294-1.928)	0.554
Poor adherence to BP medications	1.920 (1.245-2.963)	0.003	1.960 (1.195-3.214)	0.008
Non-compliance with low sodium diet	1.894 (1.01-3.606)	0.049	1.325 (0.657-2.673)	0.432
ARB	1.381 (0.745-2.560)	0.304		
ACE	1.125 (0.738-1.715)	0.584		
CCB	0.845 (0.549-1.299)	0.441		
Diuretics	1.164 (0.609-0.224)	0.646		
BB	1.272 (0.756-2.139)	0.364		

ACE: angiotensin converting enzyme, ARB: angiotensin receptor blockers, BB: beta blockers, BMI: body mass index, BP: blood pressure, CCB: calcium channel blockers, CI: confidence interval, LVH: left ventricular hypertrophy, OR: odds ratio

## Discussion

Hypertension is the most common modifiable cardiovascular risk factor in the very elderly population. Its prevalence is estimated to be more than 70% in octogenarians [9,10]. Hypertension in the elderly is characterized by an increase in SBP and a decrease in DBP, leading to an increase in PP [11]. This particularity of HTN in very elderly patients is similar to the results of our study, in which one third of patients had isolated systolic HTN and 57% of patients had an increase in PP (≥60 mmHg). Several observational studies have shown that, in a very elderly population, cardiovascular risk is directly proportional to the SBP and inversely proportional to the DBP [12]. Arterial stiffness is the main cause of the rise in SBP and PP, as well as the decline in DBP in this population [13,14]. About 51% of our octogenarian patients had uncontrolled HTN. This rate is higher than the rate reported by a French study, in which 36.6% of octogenarian had uncontrolled HTN [15]. In this study, the target BP was a SBP < 140 mmHg and a DBP < 90 mmHg, which were similar to the targets used in our study. In some therapeutic trials, including Hypertension in the Very Elderly Trial (HYVET) study [16], the target of the SBP in octogenarians was 150 mmHg. However, the 2018 European guidelines recommend prescribing antihypertensive drugs to octogenarian patients with a SBP ≥160 mmHg, with the aim of lowering SBP below 140 mmHg and DBP below 90 mmHg [17].

In our study, a third of patients had diabetes and nearly half of them had dyslipidemia. We found a significant relationship between diabetes and uncontrolled HTN (p=0.037, OR=1.66, CI95%: 1.031-2.688), but we did not find any significant association between dyslipidemia and uncontrolled HTN. The prevalences of diabetes and dyslipidemia increase with age and are higher in hypertensive patients with uncontrolled HTN [18]. Senior *et al*. reported a prevalence of diabetes of 11% in octogenarian hypertensive patients in New Zealand, however, they did not find that diabetes was correlated with BP control [19]. While the relationship between BP control and dyslipidemia is much less established in octogenarians [20]. However, treatment of dyslipidemia may be beneficial for BP levels, although clinical trials that can confirm or disprove this hypothesis are currently lacking [21]. The prevalence of chronic diseases, especially cardiovascular diseases, increases with age [22]. Age-related physiological changes, such as endothelial dysfunction and arterial stiffness, lead to an increased incidence of cardiovascular disease in the elderly. In the United States, the prevalence of coronary artery disease in octogenarians has been estimated at 32.2% in men and 18.8% of women [23] these rates are close to the rates observed in our study (30.8% in men and 15.8% in women). The prevalence of AF in our population was 14%, higher than that found in an octogenarian population in the United States (9%). Hypertension and advanced age are powerful and independent risk factors for the occurrence of nonvalvular AF [24] which explains the relatively high prevalence of AF that we found in our population.

The relationship between uncontrolled HTN and cardiovascular disease has been proven by numerous epidemiological studies in octogenarian patients [25]. These results were not confirmed in our study in which we did not find a significant association between BP control and cardiovascular disease namely coronary artery disease, AF and stroke. Renal failure is one of the most common comorbidities in very elderly and increases the risk of cardiovascular disease [26]. Its prevalence in our study was 12%. This high prevalence in this very elderly population makes them susceptible to the side effects of antihypertensive drugs, especially ACE and diuretics. These latter were used by about half of our population for ACE and 12% for diuretics. This could be the cause of ion disorders, especially dyskalemias which have been observed in 25% of our patients. The choice of drug treatment must be adapted to the clinical situation of each patient, taking into account the associated pathologies and poly-medications, particularly common in the elderly. In very elderly population, all anti-hypertensives drugs can be used, but they should be prescribed with caution. Poor adherence to anti-hypertensive drugs was relatively high in our population (41.3%) and it was significantly associated with uncontrolled HTN (p=0.008, OR=1.960, CI95%: 1.195-3.214). In very elderly patients, the prevalence of non-adherence to BP medications increases. In this group of patients, there are specific risk factors for non-adherence such as progressive cognitive decline and depression, in addition to conventional risk factors for non-adherence [27]. In our study, we found a positive correlation between non-compliance with low sodium diet and uncontrolled HTN only in univariate analysis. In very elderly patients, it is not advisable to make excessive restriction of salt because it could lead to ion disorders such as hyponatremia, undernutrition or orthostatic hypotension resulting in an increased risk of falls [28].

However, unlike to the results observed in many studies studying the effect of weight [29] and physical activity [30] on BP control in octogenarians, we did not find significant associations. Hygienic-dietary measures are essential for better BP control. In addition to reducing BP without being iatrogenic, they reduce the dose and the number of prescribed antihypertensive drugs. Lifestyle modification is recommended as a first-line treatment for all hypertensive patients, especially the elderly, where poly-medication, potential drug interactions and non-adherence to treatment are major problems in this population. This study is the first, based on the Tunisian population, to our knowledge, to report results focused on hypertensives aged 80 and over. However, several limitations of our study deserve to be mentioned. Firstly, this study did not initially plan to investigate the factors and morbidities associated with uncontrolled HTN in octogenarians, so some interesting data were not collected such as the assessment of the cognitive state of these patients. Second, the cross-sectional and observational nature of the study limits the causal association to be made with the identified correlated factors, which cannot be defined as predictors, but rather as factors associated with uncontrolled HTN. Finally, as the purpose of this study was to investigate the control of BP under the conditions of daily clinical practice, the prevalence of uncontrolled HTN may have been overestimated or underestimated because BP was measured during a single medical visit. However, this is a current methodological choice in this type of study.

## Conclusion

The results of our study show that more than half of octogenarian hypertensive patients did not reach the BP target. In this population, the presence of other comorbidities and poor adherence to BP medications are very common. We have shown that poor adherence to antihypertensive therapy is the main factor of uncontrolled HTN in these patients. Systematic research for behaviors suggesting poor medication adherence should be a priority for physicians caring for this population.

### What is known about this topic

- Hypertension is the main risk factor for most morbidities of elderly subjects, with a significant impact on their prognosis;
- Africa is not spared by this global health problem, with an annual mortality rate due to hypertension;
- Hypertension is a complicated disease that requires optimal control and persistent drug-adherence.

### What this study adds

- This is the first Tunisian study to report results focused on hypertensive patients over 80 years of age;
- More than half of octogenarian patients had uncontrolled hypertension;
- Poor adherence to anti-hypertensive drugs was very common in this population, and it was the main factor associated with uncontrolled hypertension.
